# Efficacy of hearing conservation education programs for youth and young adults: a systematic review

**DOI:** 10.1186/s12889-018-6198-7

**Published:** 2018-11-22

**Authors:** Khalid M. Khan, Sylvanna L. Bielko, Marjorie C. McCullagh

**Affiliations:** 1 Department of Environmental Health, School of Public Health, Indiana University-Bloomington, 1025 E Seventh Street, Room 025E, Bloomington, IN 47405 USA; 2 Department of Environmental Science, Public Health, and Sustainable Development, School of Applied and Natural Sciences, Taylor University, Upland, IN USA; 3 Department of Systems, Populations and Leadership, University of Michigan School of Nursing, Ann Arbor, MI USA

**Keywords:** Hearing conservation, Youth, Young adults, Technology, Educational intervention, Rural and agricultural communities, Systematic review

## Abstract

**Background:**

Many youth and young adults experience high noise exposure compounded by lack of access to hearing health education. Although the need for hearing health education programs is evident, the efficacy of these programs for youth is unclear. We evaluated the literature for efficacy of various hearing conservation programs aimed at youth and young adults, and analyzed their strengths and limitations.

**Methods:**

Studies reporting results of hearing conservation or hearing loss prevention programs with youth or young adults, using randomized controlled trials, quasi-experimental designs, experimental design, or qualitative research, and published in peer-reviewed journals in English between 2001 and 2018 were included. Studies were found through searches of selected literature databases (i.e., PubMed, Google Scholar, NIOSH Toxline, and Scopus). Identified publications were assessed for relevance, and data were extracted from the studies deemed relevant.

**Results:**

A total of 10 studies were included. Very little evidence of efficacy of hearing conservation educational programs was found in these studies. Several methodological limitations including lack of rigorous study designs, inadequate power, and application of inappropriate statistical analysis were noted. Some use of technology in programs (e.g., smartphone apps, mobile phone text messages, and computers) was observed, but conclusions as to the effectiveness of these tools were limited by the small number of studies and small sample sizes.

**Conclusions:**

The number of studies of educational hearing conservation programs for youth and young adults was low. The efficacy of the program was not reported in most studies, and it is difficult to draw public health conclusions from these studies due to their multiple methodological limitations. While use of technology in hearing conservation educational programs offers promise, its effectiveness has not been studied.

## Background

Noise from recreational activities threatens the hearing ability and health of youth and young adults around the world. In the United States, approximately 5.2 million children and adolescents, and several million young adults, experience noise-induced hearing loss [1, 2]. Non-occupational and recreational activities such as concerts, use of personal listening devices, playing musical instruments, and visiting music venues, clubs, and sporting events expose adolescents and young adults to loud noise, often resulting in transient tinnitus as an immediate impact, and irreversible hearing damage in the case of long-term exposure [3]. In these leisure activities, average sound levels commonly vary between 99.8 and 140 dB, which even after short-term exposure could negatively impact hearing ability [4–7]. In addition, millions of youth are exposed to noise in occupational settings, such as youth working on or visiting farms [8].

Although the effects of high noise on hearing (e.g., noise-induced hearing loss and tinnitus) are well-known [9, 10], high noise also affects many organ systems, resulting in a negative impact on the quality of life of the affected individuals [11]. In addition to the risks of noise-induced hearing loss and other somatic effects of noise, children exposed to high noise are at risk for academic failure and behavioral problems [12, 13]. Hearing loss affects physical and emotional functioning, social life, and employment. Importantly, hearing loss has also been associated with increased risk for injury [14]. Substantial evidence in the literature suggests that noise exposure results in heavy social and economic burdens on families and communities across various ethnic and socioeconomic groups [15].

The problem of hearing loss has been identified as high priority by leading national organizations. Healthy People 2020 includes a focus on reducing the prevalence and severity of disorders of hearing, and increasing “the proportion of adolescents aged 12 to 19 years who have ever used hearing protection devices (earplugs, earmuffs) when exposed to loud sounds or noise” [16]. The core mission and strategic plan of the National Institute for Deafness and Communication Disorders includes development of programs to prevent communication disorders [17, 18]. Noise-induced hearing loss has also been named as one of the 21 top research priorities for the century by the National Institute for Occupational Safety and Health [19], and is a high-priority area in the National Agriculture, Forestry, and Fishing Agenda [19], Occupational Safety and Health Administration [20], and the North American Guidelines for Children’s Agricultural Tasks [21].

Use of hearing protectors, maximizing the distance from the source of noise, and providing enclosure to minimize noise transmission are a few methods of mitigating the effects of high noise exposure in occupational and recreational settings [22]. However, educational programs to promote use of these and other hearing conservation approaches among youth and young adults are few in number [23–28]. Also, data gaps exist regarding the efficacy of technology in reducing negative effects of hazardous noise exposure. In different public health intervention studies, promotion of positive health behavior among adolescents and farm youth were effectively accomplished through the use smartphone apps, mobile text messages, and computer and web-based educational programs [29–31]. The purpose of this systematic review was to a) review the efficacy of hearing conservation programs aimed at youth and young adults, and b) analyze their weaknesses and limitations.

## Methods

Methods for the systematic literature review were adapted from PRISMA guidelines [32] and included the following steps: search for studies, assess relevance of identified publications, data extraction, categorization of studies, and synthesis of results from included studies. The following inclusion criteria were applied: studies that included hearing conservation or hearing loss prevention programs with youth or young adults; AND studies that used randomized controlled trials, quasi-experimental designs, experimental design, OR qualitative research; AND studies published in peer-reviewed journals in English between 2001 and 2017.

Studies were found through searches of selected literature databases (i.e., PubMed, Google Scholar, NIOSH Toxline, and Scopus) using combinations of the following keywords: “hearing protection intervention” OR “hearing conservation intervention” OR “hearing protection” OR “hearing protection conservation” OR “hearing conservation” OR “hearing” OR “intervention” OR “noise induced hearing loss” AND “agricul*” OR “rural” OR “noise” AND “technology” AND “smartphone” OR “computer” AND “internet” AND “text messaging” AND “school based.” Multiple combinations of keywords were used to ensure that all relevant studies were discovered. Searches were conducted from October through December 2017. This resulted in a total of 23,306 reports (duplicates included), which were distributed between 6982 hits in PubMed, 16,293 hits in Google Scholar, 7 hits in NIOSH Toxline, and 24 hits in Scopus. A total of 7004 unique reports were screened by title and abstract, and out of those, 20 underwent full article screening. Ten studies met all inclusion criteria as listed above.

The searches were conducted independently by two authors (KMK and SLB) in consultation with a librarian at Indiana University Bloomington who had expertise in scientific literature search in public health. Titles and abstracts were read independently, and relevance was assessed based upon the inclusion criteria. All authors participated in the full text review where the full article was read and discussed. All relevant studies were included, irrespective of their scientific quality. Information regarding study design, interventions conducted, study objective, randomization of subjects, data collection, analyses performed, and relevant results were extracted from reports.

## Results

**Fig. 1:**
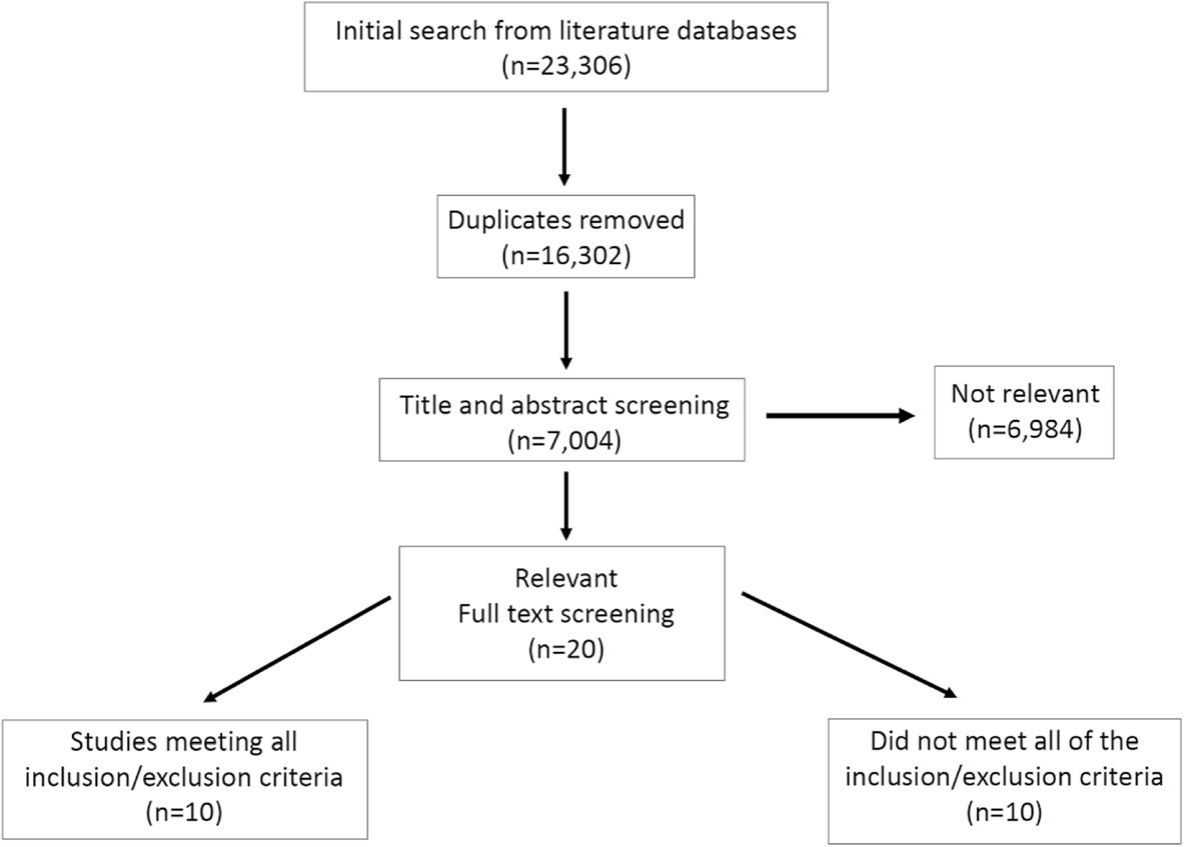
Selection Process for Identification of the Studies for Review

As indicated in Fig. 1, a total of 20 publications were initially identified as “relevant” after screening the titles and abstracts of 7004 publications. These 20 studies addressed topics related to hearing conservation or hearing loss prevention among youth and young adults. However, 10 studies did not meet all the inclusion criteria and therefore, the remaining 10 publications conducted in two countries (8 in the USA, and 2 in Belgium) were finally selected for this review. Table 1 provides a summary of characteristics of the study including study objectives, sample characteristics, study groups that were compared and instruments for outcome measures. The selected research reports, published over the past 18 years (2001–2018), employed a variety of study designs including cluster-randomized controlled trials (*n* = 3), quasi-experimental studies with crossover design with or without a control group (*n* = 4), and experimental studies with multiple groups (*n* = 3).

**Table 1:** Summary of Selected Characteristics of Reviewed Studies (*n* = 10)

Publication country	Study design	Study objective	Sample eligibility of participants	How participants were allocated to groups	Use of control group(s)	Instruments for outcome measurement
Reed et al. (2001) [37] USA	Quasi-experimental crossover design	To test the effectiveness of two sets of instructional materials on farm safety designed through participatory action research involving teachers and students. AgDARE experiential learning curriculum on four disabilities including hearing loss was used. AgDARE used stories told by farmers with disability because of poor safety behavior.	Adolescents aged 14–16 years (9th and 10th graders) from 21 high schools located in rural and agricultural communities in three states (*n* = 790).	Two intervention groups had 373 participants and 417 were enrolled in the control group.	Yes	Students completed the Farm Safety Attitude (FSA) instrument and the Stages of Change (SOC) at both pre and one year post ntervention phases
Lee et al. (2004) [36] USA	Cluster randomized controlled trial	To evaluate a rural health & safety initiative implemented in 4000 National FFA (formerly Future Farmers of America) Chapters across the United States.	Rural high school students from 123 FFA chapters (*n* = 3081)	Standard intervention, enhanced intervention and control groups had 1059, 683 and 1339 participants respectively.	Yes	Students reported safety knowledge, safety consciousness, self-reported leadership, dangerous risk-taking, self-esteem, safety campaign participation, injury experiences at pre and academic year 1 and 2 post-intervention.
Joseph et al. (2007) [38] USA	Quasi-experimental without control (randomization & manipulation; controlled behavior intervention trial)	To compare the effect of small-group training on the attenuation performance of passive insert-type HPDs with individual training. To compare the results of formal training with no training.	College students from both rural and urban communities (*n* = 100); demographic characteristics not well-defined.	Each of the four HPD training groups had 25 participants who were randomly assigned into groups.	No	Participants were evaluated for real-ear attenuation at threshold (REAT) at third-octave noise band between 125 and 8000 Hz. REAT measurements were augmented by the use of Hearing Loss Prevention Attitude-Belief (HLPAB) survey.
Berg et al. (2009) [33] USA	Cluster randomized controlled trial	To determine whether a hearing conservation program for youth involved in farm work resulted in (i) reduced prevalence of NIHL at 3-year follow up and/or (ii) an increased use of HPD when compared with controls.	Rural school students enrolled in 7th to 9th grades and actively and regularly involved in farm tasks (*n* = 753).	Intervention and control groups had 378 and 375 students respectively from 34 schools at baseline although 690 were available at follow-up.	Yes	Primary outcome measures were audiometric threshold changes from baseline to the 3-year follow-up whereas secondary outcome measure was self-reported use of HPD using a three point Likert-type scale (never, sometimes, and always).
Kotowski et al. (2011) [39] USA	Randomized experimental study with two group post-test only design	To determine if brochures developed on the risks of NIHL using the Extended Parallel Process Model (EPPM) increased intentions to use HPDs for college students.	Undergraduate college students exposed to noises from sporting and recreational sources (*n* = 176). Study setting (i.e. urban or rural) remained undefined.	Subjects were randomly put into intervention (*n* = 93) and control (*n* = 83) conditions.	Yes	Participants reported perceptions of hearing loss threat, efficacy to use earplugs and intentions to use earplugs when in loud environments.
Marlenga et al. (2011) [24] USA	Cluster randomized controlled trial	To assess whether a hearing conservation program for youth involved in farm work resulted in reduced prevalence of NIHL and sustained HPD use compared with a concurrent control group at the 16-year follow-up period.	Young adults (at 16-year follow-up) who were high or middle school students located in rural and agricultural communities at baseline (*n* = 392).	Children from 17 intervention school and equal number of control schools were available at the follow-up. Intervention and control groups had 200 and 192 participants respectively during follow-up data collection.	Yes	Self-reported use of HPD for each recreational and occupational categories was used as the primary outcome measures. Audiometric outcome measures were threshold changes from baseline to 16-year follow-up in (1) individual frequencies, (2) OSHA standard threshold shift, (3) low frequency average, (4) high-frequency average, and (5) the bulge depth statistic.
Martin et al. (2013) [27] USA	Experimental study with randomized selection of groups	To evaluate the sustainable impacts of four different types of interpersonal and interactive educational interventions on NIHL prevention from baseline to immediate post-intervention and follow-up.	Children from 53 fourth grade classrooms participated in the study (*n* = 1120). Schools were located in communities with high-minority and under-represented populations.	Classrooms were randomly placed into four experimental and one no-intervention groups. Number of classrooms (children) analyzed: older peer 13 (272), health educator 10 (209), on site museums 9 (185), web-based 15 (322), and no-intervention 6 (125).	Yes	Knowledge, attitudes and intended behaviors regarding sound exposure and use of appropriate hearing protection strategies were the outcome measures assessed via questionnaire survey.
Gilles et al. (2014) [34] Belgium	Quasi-experimental one-group pre/post test	To examine if a preventive campaign can alter attitudes toward noise and enhance the use of HPD among adolescents.	Flemish high school students (*n* = 547) exposed to high levels of recreational noise. Study setting (i.e. urban or rural) remained undefined.	Not applicable as only one group was observed before and after intervention.	No	Youth attitudes and beliefs were assessed by the use of youth attitudes toward noise scale (YANS) and the beliefs about hearing protection and hearing loss (BAHPHL) questionnaire respectively. Self-reported hearing protection was also recorded.
Keppler et al. (2015) [40] Belgium	Experimental study with one group	To evaluate the effects of a hearing education program on recreational noise exposure, attitudes and beliefs toward noise, hearing loss, and HPD use after approximately 6 months following training.	Young adults (*n* = 78) exposed to loud recreational noise. Study setting (i.e. urban or rural) was not defined.	Not applicable as only one group was observed at pre and post training.	No	Hearing status was determined by admittances measures, pure-tone audiometry, and registration of otoacoustic emissions (OAEs).
Khan et al. (2018) [35] USA	Experimental study with cluster randomization	To evaluate the efficacy of low-cost technology-based approaches to enhance hearing conservation knowledge and attitude and the use of HPD among adolescent farm workers.	High school students living in rural and agricultural communities (*n* = 70).	Six schools were divided into three clusters and then each cluster randomly received a specific format of intervention. Adolescents were randomly recruited from each school. Fifty students from three groups were available at six-week post intervention.	No	Self-reported hearing conservation knowledge, attitude and use of hearing protection during noisy tasks in agriculture.

*NIHL* Noise-induced hearing loss, *HPD* Hearing protection device

### Study settings and participant characteristics

Studies included in this literature review demonstrated wide variations in study settings and subject characteristics (e.g., age, education levels). There were six studies of high-school children [24, 33–37], two of college-aged youth [38, 39], and one each of elementary school children [27] and young adults [40]. Sites included schools or colleges [27, 35, 36, 38, 39] and farms [24, 33, 37]; in one study no information about the study setting was provided [40]. Half of the studies in this review were conducted in agricultural and/or rural communities, mainly with children enrolled in agricultural classes [24, 33, 35–37]. Three studies did not provide clear information about the geographical characteristics of the sample (i.e. rural or urban) [34, 39, 40], although occupational characteristics of the participants indicated that subjects living rural or semi-urban were included in the study sample. One study recruited college students from both urban and rural areas [38], whereas another urban study relied on populations where a significant number of children came from minority and under-represented families in semi-urban or rural areas [27].

### Features of interventions

#### Types of interventions

Interventions were exclusively educationally-based, directed at increasing users’ knowledge of noise and hearing, with an implicit goal of influencing hearing conservation behavior. Environmental modification and policy development approaches were not represented. Educational content on sources of loud noise, enhancing positive attitudes towards hearing conservation, noise-induced hearing loss, and use of hearing protection were the main focus areas for content of education in the selected studies. In two US studies in agricultural communities, hearing conservation was nested in health and safety education programs or curricula that also provided education to high school students on other farm safety hazards [36, 37]. Other studies were conducted with a narrower aim toward reducing the risk of noise-induced hearing loss.

#### Educational methods

In a limited number of studies, researchers employed various types of teaching and learning methods to accomplish the goals of the intervention projects. Most of these methods were interactive and conducted in field settings. Notable teaching/learning methods included hands-on demonstration of fitting of hearing protection devices [38], interactive learning and training via face-to-face and computer training [27, 33, 35], communication of the results of clinical evaluation of hearing status of the participants [40], and the use of a model of persuasive communication [39].

### Efficacy of educational hearing conservation programs

Findings regarding efficacy of programs are summarized in Table 2. Descriptions of intervention approaches often lacked detail. For example, one study that examined the effect of a government-sponsored preventive campaign on noise exposure for high school students (via TV and radio commercials and social media), failed to include a description of the informational content disseminated [34]. Only two studies demonstrated statistically significant efficacy, i.e., results showed significant improvement in the frequency of hearing protection use in the intervention group when compared with the control group [24, 37]. Additionally, none of the studies provided information about the roles of the educators in implementing the program.

**Table 2:** Characteristics of Interventions and Major Findings

Publication	Intervention intended for each group of youth	Findings on the efficacy of interventions	Limitations in methodology and data interpretation
Reed et al. (2001) [37]	1) Narrative simulations based on stories on farm activities and 2) Simulations of farm tasks while students pretended to have a disability	AgDARE curriculum improved safety attitude and behavior	• Long-term impact on hearing protection use was examined only in small a subset of adolescents. • Control group was older than the intervention groups. • Not a clinical trial. • Only safety attitude changes were measured but change of HPD use was not mentioned.
Lee et al. (2004) [36]	1) Standard intervention group participated in 10 educational activities on farm safety including interactive training, printed and electronic materials, discussions on activities in national forum and writing information for newsletter and 2) Enhanced intervention group participated in all standard activities and additionally received telephone calls, mailings, personal contacts and free personal protective equipment.	Nationwide program based in FFA did not result in changes in agricultural safety knowledge, attitudes, leadership, self-concept, or injuries. Program also failed to develop sustainable community partnership.	• Intervention fidelity was poor. • Initial positive outcomes were not retained at 1 year. • No process measures observed in control group.
Joseph et al. (2007) [38]	1) Small-group training on the attenuation performance of formable HPD, 2) small-group training on the attenuation performance of premolded HPD, 3) individual training on the attenuation performance of formable HPD and 4) individual training on the attenuation performance of premolded HPD.	Both group and individual training formats demonstrated significant effect both types of HPDs on attenuation and attitude but the difference in attenuation between group and individual training was not significant.	• There was no follow-up data collection and therefore, no evidence of sustained learning was obtained.
Berg et al. (2009) [33]	Hearing conservation program comprised of (i) classroom instruction at each school, (ii) distribution of HPD, (iii) direct mailings to the student’s home, (iv) noise level assessments at the student’s home, and (v) yearly audiometric testing.	Students in the hearing conservation intervention group reported more frequent HPD use but no post-intervention evidence of reduced levels of NIHL was observed.	• Since NIHL is cumulative, a 3-year study was likely not long enough to evaluate the efficacy of this intervention. • The report lacked description of control group activities.
Kotowski et al. (2011) [39]	Brochure developed on the threats, severity and susceptibility of NIHL and efficacy of behaviors that can minimize the threats.	Viewing brochure improved perception of NIHL and efficacy to use earplugs without changing intention of HPD use.	• Convenience sampling approach was used • Lacked statistical analysis as confounding variables were not taken into account.
Marlenga et al. (2011) [24]	Described above*.	Participants from the intervention group reported significantly higher use of HPDs in agricultural activities and greater use of HPDs for shooting guns than the controls. For other activities, both groups reported similar uses of HPDs. No significant differences between groups with respect to objective measures of NIHL was observed.	• The study lacked power when compared with parent study*. • Low enrollment rate at follow-up (i.e. 52% of the subjects was retained). • The study demonstrated limited effectiveness in preventing early NIHL in rural high school students at 16-year follow up.
Martin et al. (2013) [27]	1) Classroom presentation by older-peer educators (high school students), 2) classroom presentation by health professional educators (school nurses), 3) on-site museum visits interaction with a museum exhibition on NIHL and tinnitus prevention, and 4) virtual museum experience via internet.	Positive effects in knowledge, attitude and behavior in all formats of interventions were observed. In terms of effectiveness, the classroom programs were more effective than the internet-based virtual exhibit, which was more effective than the visit to the museum exhibition. Interpersonal, interactive educational interventions such as the classroom program are more effective and have longer impact than self-directed learning experiences for NIHL and tinnitus prevention.	• Detail information about the process of randomization was not provided. • Duration (i.e. time spent) of the four different formats of intervention varied. • Validity of the knowledge questions was not addressed. • The study did not detect significant differences between groups perhaps due to weak statistical power. • No adjustment for sociodemographic variables was observed.
Gilles et al. (2014) [34]	A governmental prevention campaign ‘Anything less is the max’ targeting high school students via television and radio commercials, social media, posters and interactive website was used. Major emphasis of the intervention was placed on loud music, controlled use of personal listening devices and prevention approaches at other noisy situations.	Scores on the youth attitudes towards noise scale (YANS) and the beliefs about hearing protection and hearing loss (BAHPHL) decreased significantly after intervention. Hearing protection use increased significantly from pre to post intervention. Use of personal listening devices did not change.	• Weak study design as there was no comparison or control group. • Effect sizes not reported. • Random sampling strategy was not used. • Time of post-testing was not specified.
Keppler et al. (2015) [40]	This education program was presented one-on-one between the audiologist and the subject using a structured slide show. It contained information about functioning of the normal auditory system, the effects of noise exposure on the auditory system, and the preventative measures including information regarding HPDs. Five questions were asked to the participants to evaluate the level of understanding. Audiometric evaluation was also performed on the subjects.	Educational intervention improved the hearing protection attitude, belief and frequency of HPD use. There was a significant decrease in recreational noise exposure between pre and post training sessions.	• Study was conducted with a very small sample size (i.e. pilot study). • It used a wide range of time between pre and post intervention data collection for the participants.
Khan et al. (2018) [35]	1) Classroom training on noise exposure and HPD use, 2) classroom training on noise exposure and HPD use coupled with smartphone app training to measure noise levels during noisy farm tasks and 3) computer training on on noise exposure and HPD use.	All three formats of educational interventions improved hearing conservation and protection knowledge, attitude & HPD use within each group. When the groups were compared the changes of knowledge, attitude & HPD use between groups were non-significant.	• Study was conducted with a very small sample size (i.e. pilot study). • Very short follow-up period was used and therefore, the sustained effects of interventions could not be measured. • Validity of instruments used to measure outcome variables was not reported.

*NIHL* Noise-induced hearing loss, *HPD* Hearing protection device

*Berg et al. (2009) [33]

The use of technologies in educating youth about noise exposure and noise-induced hearing loss prevention was addressed in a few studies [27, 34, 35, 38]. In the Dangerous Decibels program, an internet-based virtual museum experience was designed to enhance hearing conservation knowledge among fourth grade students [27]. In this virtual experience, the user navigates the virtual exhibit to explore ear structures, sound waves, and hearing protection strategies. The virtual exhibition can be adapted into multiple languages. The only drawback of this educational tool was the short effect of training, which failed to sustain intent to use hearing protectors for more than 3 months [27]. In another study of adolescent high school students, the risk of loud music and use of hearing protection in noisy environment were effectively mitigated via multiple strategies such as television and radio commercials, social network sites, and an educational website [34]. One limitation of this large countrywide program was the high cost of the campaign. In a pilot study, an hour-long computer training on hearing conservation in agriculture was developed targeting adolescent farmworkers who demonstrated improved hearing protection knowledge, attitude and use of hearing protection [35]. A software program was used to break information into smaller units, required mastery of the material before moving from one unit to the next, was self-paced, and included pre- and post-tests and quizzes throughout the training to monitor accomplishment of learning objectives. The same study also found knowledge of the use of smartphone noise apps significantly improved hearing protection behavior among adolescents [35]. An audiovisual training illustrating the process of correct insertion of hearing protection devices was found useful for older youth such as college students, although it was used as a small component of a hearing loss prevention training [38]. Trainees liked the fact that the training video could be viewed at their convenience and more than once if necessary.

### Limitations in study design and data analysis

Several limitations and weaknesses were observed in study design and data analysis (Table 2). There were several studies with small sample sizes (*n* ≤ 100) that lacked adequate statistical power to detect group differences [35, 38–40]. Wide variations in the length of follow-up were also observed. While some studies used long-term follow-up ranging from one to 3 years [36] or even longer [24], most studies used very short-term follow-up such as few months [27] or even weeks [35]. Follow-up data on the study subjects were not reported in one study, therefore failing to report on the effect of the program [38]. In two one-group quasi-experimental studies that compared pre- and post-intervention measures, the timing of post-testing was not specified [34], or the duration between these two waves of data collection varied widely across the participants of the study [40]. Only three studies used a randomized controlled trial design, the most robust and highly recommended study design to evaluate the effectiveness of interventions [24, 33, 36]. Two other studies followed a randomized approach to assigning subjects to experimental groups, however, they did not meet all the criteria of a randomized trial due to other methodological limitations, such as lack of control group [38] or absence of pre- versus post-intervention comparison [39]. The randomization process was not described in one study [27]. Absence of control group was also noticed in three experimental studies [34, 35, 40], among which only one study followed a cluster randomization approach to assign three different types of training formats to the experimental groups [35]. In one study, non-randomized selection of comparison groups resulted in an older control group [37]. In several studies, evidence of the validity and reliability of instruments was lacking [35, 36, 40]. Methodological limitations were also evident in the statistical analysis and reporting of data. Some noticeable weaknesses included lack of adjustment for sociodemographic confounding variables [27, 39], non-reporting of effect size [34], and lack of application of appropriate statistical analysis for determining the effects of intervention, perhaps due to small sample sizes.

## Discussion

This review used a systematic and sensitive search strategy, together with multiple literature databases, to ensure that all studies meeting inclusion criteria were reviewed. Overall, we found a small number of hearing health intervention studies for youth with multiple methodological limitations. In addition to problems with study designs, the quality of reporting was low. Many studies failed to include essential elements needed to assess trial quality, e.g., robust design, presence of sufficient statistical information, specification of eligibility criteria, and random allocation of subjects [41]. As a result, the reports lack critical information to inform future programs designed to promote hearing health.

In the last 15 years, there have been only two previously published reviews of hearing conservation programs for youth; the most recent 5 years ago. Our review included some of the same studies included in previous reviews, but differed in quality. For example, we included a critical analysis of study methods and efficacy, which were not included in the previous review papers. Furthermore, we expanded the inclusion criteria to include a greater breadth of study populations and study settings compared to previous reviews [42, 43]. In addition, we included a review of study strengths and weaknesses, as well as recommendations for future study design and policy development.

Despite the recognition of noise exposure as a priority occupational health problem, our extensive search of literature from 2001 onward produced only 10 reports that met study inclusion criteria. Weaknesses in study design and methods were common among reviewed studies, e.g., small sample sizes, lack of rigorous study designs (e.g., randomized controlled trials), inadequate power, and application of inappropriate statistical analysis. These limitations seriously impaired the validity of study conclusions and generalizability of results. While several intervention studies included in the review demonstrated improvement in hearing conservation knowledge, attitude, and/or behavior from pre to post intervention, most post-tests were short-term (a few weeks or months, or none at all); long-term and sustained effects of such interventions were rare. There is a need for high-quality studies using robust trial designs to test longer-term effectiveness of hearing conservation programs for youth and young adults. We recommend that reports of intervention studies should follow commonly-accepted protocols for trial reporting, such as CONSORT and PEDro [41].

There was wide variation in study settings, outcome measures, instruments, and intervention approaches across studies. Similarly, intervention methods were diverse: school curriculum on hearing conservation, distribution of printed materials (e.g., brochures, flyers), use of social media and the internet, face-to-face motivational activities, group learning, and technological approaches such as smartphone apps, computer training, and text message reminders.

The role of technology in promoting many types of health behavior is of great interest. Among youth, several studies employing technology-based program have demonstrated high levels of efficacy with multiple health behaviors (e.g., nutrition and obesity, sexual and mental health, cancers, and asthma) [44–50]. Furthermore, several studies [51, 52] have shown that youth are more inclined to educate themselves via computer training and internet-based tools, and do so more effectively and sustainably when compared with traditional formats of education such as reading materials and lecture-format training. Technology-based programs also offer an advantage over traditional training methods because they offer interactive materials for active learning. Results of this review indicate that electronic technologies are promising tools in educating a widely dispersed audience including low-income rural and agricultural communities. We observed that training offered via computer software or internet could create opportunities for self-paced instructions allowing the participants move from one module to the next after clear understanding on the topics presented. On the other hand, radio and television commercials and mobile phone text messages can briefly but efficiently transmit the core knowledge in the forms of prompts and reminders. The availability of low-cost or free smartphone applications for noise measurement could also contribute to youth decision-making about the need for and selection of hearing protection devices and other noise-control strategies. Initial large investment required to develop the technology-based learning materials can be easily compensated by a number of sustained learning outcomes in diverse target populations.

## Conclusions

We reviewed the efficacy of hearing conservation interventions aimed at youth and young adults; analyzed the weaknesses and limitations of published studies, generated recommendations for future development of hearing conservation programs, including use of technology (e.g., smartphone apps, mobile phone text messages, and computers).

The use of technology in promoting hearing conservation is a promising recent development in hearing conservation for youth. Although few studies to date have used technology in hearing conservation programs [27, 34, 35, 38], technology-based interventions have potential for reaching larger and difficult-to-reach audiences, and in cost-effective ways. Therefore, health interventionists and program designers may consider integrating technology-based educational approaches in future hearing conservation programs targeting noise-exposed youth, particularly those who are geographically dispersed (e.g., rural areas, small-towns, and farming communities) and may otherwise lack access to quality hearing conservation programs.

## Abbreviations

dB: Decibel
HPD: Hearing protection device
NIHL: Noise-induced hearing loss
NIOSH: National Institute of Occupational Safety and Health
RCTs: Randomized controlled trials
